# Supplementary material for: The Effectiveness of E-Health Interventions Promoting Physical Activity and Reducing Sedentary Behavior in College Students: A Systematic Review and Meta-Analysis of Randomized Controlled Trials
Source: Int J Environ Res Public Health. 2022 Dec 25;20(1):318. doi: 10.3390/ijerph20010318 (PMC9819541; doi:10.3390/ijerph20010318)
Supplement: Supplementary file 1 [file ijerph-20-00318-s001.zip › Search strategy .pdf]

Search strategy

PubMed Searching:

Included: 65; Search Name: e-health for pa and sb; Date Run: 22/06/2022

Search: ((((((((((((college student[Title/Abstract]) OR (college students[Title/Abstract])) OR (university student[Title/Abstract])) OR (university students[Title/Abstract])) OR (undergraduate[Title/Abstract])) OR (undergraduates[Title/Abstract])) OR (tertiary school student[Title/Abstract])) OR (tertiary school students[Title/Abstract])) OR (higher school student[Title/Abstract])) OR (higher school students[Title/Abstract])) AND (((randomized controlled trial[Publication Type]) OR (randomized[Title/Abstract])) OR (placebo[Title/Abstract]))) AND (("Telemedicine"[Mesh]) OR (((((((((((((((((((((((((((((((((((ehealth[Title/Abstract]) OR (smartphone application[Title/Abstract])) OR (smartphone app[Title/Abstract])) OR (mobile app[Title/Abstract])) OR (mobile application[Title/Abstract])) OR (app[Title/Abstract])) OR (mobile phone[Title/Abstract])) OR (smartphone*[Title/Abstract])) OR (mobile device[Title/Abstract])) OR (PDA[Title/Abstract])) OR (tablet[Title/Abstract])) OR (cell phone[Title/Abstract])) OR (text message*[Title/Abstract])) OR (sms[Title/Abstract])) OR (short message service[Title/Abstract])) OR (mobile health[Title/Abstract])) OR (mHealth[Title/Abstract])) OR (m-health[Title/Abstract])) OR (internet[Title/Abstract])) OR (telehealth[Title/Abstract])) OR (telemedicine[Title/Abstract])) OR (eHealth[Title/Abstract])) OR (e-health[Title/Abstract])) OR (iPod[Title/Abstract])) OR (Fitbit[Title/Abstract])) OR (Garmin[Title/Abstract])) OR (Jawbone[Title/Abstract])) OR (Nike[Title/Abstract])) OR (ambulatory monitoring[Title/Abstract])) OR (ambulatory assessment[Title/Abstract])) OR (wireless technology[Title/Abstract])) OR (accelerometer[Title/Abstract])) OR (pedometer[Title/Abstract])) OR (wearable*[Title/Abstract])) OR (wearable activity tracker[Title/Abstract])))) AND ((((((((((((("Exercise"[Mesh]) OR ((((((((((((((((((((((((((exercise[Title/Abstract]) OR (Exercises[Title/Abstract])) OR (Physical Activity[Title/Abstract])) OR (Activities, Physical[Title/Abstract])) OR (Activity, Physical[Title/Abstract])) OR (Physical Activities[Title/Abstract])) OR (Exercise, Physical[Title/Abstract])) OR (Exercises, Physical[Title/Abstract])) OR (Physical Exercise[Title/Abstract])) OR (Physical Exercises[Title/Abstract])) OR (Acute Exercise[Title/Abstract])) OR (Acute Exercises[Title/Abstract])) OR (Exercise, Acute[Title/Abstract])) OR (Exercises, Acute[Title/Abstract])) OR (Exercise, Isometric[Title/Abstract])) OR (Exercises, Isometric[Title/Abstract])) OR (Isometric Exercises[Title/Abstract])) OR (Isometric Exercise[Title/Abstract])) OR (Exercise, Aerobic[Title/Abstract])) OR (Aerobic Exercise[Title/Abstract])) OR (Aerobic Exercises[Title/Abstract])) OR (Exercises, Aerobic[Title/Abstract])) OR (Exercise Training[Title/Abstract])) OR (Exercise Trainings[Title/Abstract])) OR (Training, Exercise[Title/Abstract])) OR (Trainings, Exercise[Title/Abstract])))) OR ("Resistance Training"[Mesh])) OR (((((((((((((((((((((((Training, Resistance[Title/Abstract]) OR (Strength Training[Title/Abstract])) OR (Training, Strength[Title/Abstract])) OR (Weight-Lifting Strengthening Program[Title/Abstract])) OR (Strengthening Program, Weight-Lifting[Title/Abstract])) OR (Strengthening Programs, Weight-Lifting[Title/Abstract])) OR (Weight Lifting Strengthening Program[Title/Abstract])) OR (Weight-Lifting Strengthening Programs[Title/Abstract])) OR (Weight-Lifting Exercise Program[Title/Abstract])) OR (Exercise Program, Weight-Lifting[Title/Abstract])) OR (Exercise Programs, Weight-Lifting[Title/Abstract])) OR (Weight Lifting Exercise Program[Title/Abstract])) OR (Weight-Lifting Exercise Programs[Title/Abstract])) OR (Weight-Bearing Strengthening Program[Title/Abstract])) OR (Strengthening Program, Weight-Bearing[Title/Abstract])) OR (Strengthening Programs, Weight-Bearing[Title/Abstract])) OR (Weight Bearing Strengthening Program[Title/Abstract])) OR (Weight-Bearing Strengthening Programs[Title/Abstract])) OR (Weight-Bearing Exercise Program[Title/Abstract])) OR (Exercise Program, Weight-Bearing[Title/Abstract])) OR (Exercise Programs, Weight-Bearing[Title/Abstract])) OR (Weight Bearing Exercise Program[Title/Abstract])) OR (Weight-Bearing Exercise Programs[Title/Abstract]))) OR ("Physical

Endurance"[Mesh])) OR (((Stamina, Physical[Title/Abstract]) OR (Physical Stamina[Title/Abstract])) OR (Endurance, Physical[Title/Abstract]))) OR ("Muscle Stretching Exercises"[Mesh])) OR ((((((((((((((((((((((Exercises, Muscle Stretching[Title/Abstract]) OR (Exercise, Muscle Stretching[Title/Abstract])) OR (Muscle Stretching Exercise[Title/Abstract])) OR (Static Stretching[Title/Abstract])) OR (Stretching, Passive[Title/Abstract])) OR (Passive Stretching[Title/Abstract])) OR (Relaxed Stretching[Title/Abstract])) OR (Stretching, Relaxed[Title/Abstract])) OR (Static-Passive Stretching[Title/Abstract])) OR (Static Passive Stretching[Title/Abstract])) OR (Stretching, Static-Passive[Title/Abstract])) OR (Isometric Stretching[Title/Abstract])) OR (Stretching, Isometric[Title/Abstract])) OR (Active Stretching[Title/Abstract])) OR (Stretching, Active[Title/Abstract])) OR (Static-Active Stretching[Title/Abstract])) OR (Static Active Stretching[Title/Abstract])) OR (Stretching, Static-Active[Title/Abstract])) OR (Ballistic Stretching[Title/Abstract])) OR (Stretching, Ballistic[Title/Abstract])) OR (Dynamic Stretching[Title/Abstract])) OR (Stretching, Dynamic[Title/Abstract])) OR (Proprioceptive Neuromuscular Facilitation (PNF) Stretching[Title/Abstract]))) OR ("Walking"[Mesh])) OR ((walk[Title/Abstract]) OR (ambulation[Title/Abstract]))) OR ("Sedentary Behavior"[Mesh])) OR (((((((((Behavior, Sedentary[Title/Abstract]) OR (Sedentary Behaviors[Title/Abstract])) OR (Sedentary Lifestyle[Title/Abstract])) OR (Lifestyle, Sedentary[Title/Abstract])) OR (Physical Inactivity[Title/Abstract])) OR (Inactivity, Physical[Title/Abstract])) OR (Lack of Physical Activity[Title/Abstract])) OR (Sedentary Time[Title/Abstract])) OR (Sedentary Times[Title/Abstract])) OR (Time, Sedentary[Title/Abstract])))

Coachrane Searching:277

Included: 277; Search Name: e-health for pa and sb; Date Run: 22/06/2022

#1 : MeSH descriptor: [Exercise] explode all trees

#2 : (Exercises):ti,ab,kw OR (Physical Activity):ti,ab,kw OR (Activities, Physical):ti,ab,kw OR (Activity, Physical):ti,ab,kw OR (Physical Activities):ti,ab,kw OR (Exercise, Physical):ti,ab,kw OR (Exercises, Physical):ti,ab,kw OR (Physical Exercise):ti,ab,kw OR (Physical Exercises):ti,ab,kw OR (Acute Exercise):ti,ab,kw OR (Acute Exercises):ti,ab,kw OR (Exercise, Acute):ti,ab,kw OR (Exercises, Acute):ti,ab,kw OR (Exercise, Isometric):ti,ab,kw OR (Exercises, Isometric):ti,ab,kw OR (Isometric Exercises):ti,ab,kw OR (Isometric Exercise):ti,ab,kw OR (Exercise, Aerobic):ti,ab,kw OR (Aerobic Exercise):ti,ab,kw OR (Aerobic Exercises):ti,ab,kw OR (Exercises, Aerobic):ti,ab,kw OR (Exercise Training):ti,ab,kw OR (Exercise Trainings):ti,ab,kw OR (Training, Exercise):ti,ab,kw OR (Trainings, Exercise):ti,ab,kw

#3: MeSH descriptor: [Resistance Training] explode all trees

#4: (Training, Resistance):ti,ab,kw OR (Strength Training):ti,ab,kw OR (Training, Strength):ti,ab,kw OR (Weight-Lifting Strengthening Program):ti,ab,kw OR (Strengthening Program, Weight-Lifting):ti,ab,kw OR (Strengthening Programs, Weight-Lifting):ti,ab,kw OR (Weight Lifting Strengthening Program):ti,ab,kw OR (Weight-Lifting Strengthening Programs):ti,ab,kw OR (Weight-Lifting Exercise Program):ti,ab,kw OR (Exercise Program, Weight-Lifting):ti,ab,kw OR (Exercise Programs, Weight-Lifting):ti,ab,kw OR (Weight Lifting Exercise Program):ti,ab,kw OR (Weight-Lifting Exercise Programs):ti,ab,kw OR (Weight-Bearing Strengthening Program):ti,ab,kw OR (Strengthening Program, Weight-Bearing):ti,ab,kw OR (Strengthening Programs, Weight-Bearing):ti,ab,kw OR (Weight Bearing Strengthening Program):ti,ab,kw OR (Weight-Bearing Strengthening Programs):ti,ab,kw OR (Weight-Bearing Exercise Program):ti,ab,kw OR (Exercise Program, Weight-Bearing):ti,ab,kw OR (Exercise Programs, Weight-Bearing):ti,ab,kw OR (Weight Bearing Exercise Program):ti,ab,kw OR (Weight-Bearing Exercise Programs):ti,ab,kw

#5: MeSH descriptor: [Physical Endurance] explode all trees

#6: (Stamina, Physical):ti,ab,kw OR (Physical Stamina):ti,ab,kw OR (Endurance, Physical):ti,ab,kw

#7: MeSH descriptor: [Muscle Stretching Exercises] explode all trees

#8 : (Exercise, Muscle Stretching):ti,ab,kw OR (Exercises, Muscle Stretching):ti,ab,kw OR (Muscle Stretching Exercise):ti,ab,kw OR (Static Stretching):ti,ab,kw OR (Stretching, Passive):ti,ab,kw OR (Passive Stretching):ti,ab,kw OR (Relaxed Stretching):ti,ab,kw OR (Stretching, Relaxed):ti,ab,kw OR (Static-Passive Stretching):ti,ab,kw OR (Static Passive Stretching):ti,ab,kw OR (Stretching, Static-Passive):ti,ab,kw OR (Isometric Stretching):ti,ab,kw OR (Stretching, Isometric):ti,ab,kw OR (Active Stretching):ti,ab,kw OR (Stretching, Active):ti,ab,kw OR (Static-Active Stretching):ti,ab,kw OR (Static Active Stretching):ti,ab,kw OR (Stretching, Static-Active):ti,ab,kw OR (Ballistic Stretching):ti,ab,kw OR (Stretching, Ballistic):ti,ab,kw OR (Dynamic Stretching):ti,ab,kw OR (Stretching, Dynamic):ti,ab,kw OR (Proprioceptive Neuromuscular Facilitation (PNF) Stretching):ti,ab,kw

#9: MeSH descriptor: [Walking] explode all trees

#10: (walk):ti,ab,kw OR (ambulation):ti,ab,kw

#11: MeSH descriptor: [Sedentary Behavior] explode all trees

#12: (Behavior, Sedentary):ti,ab,kw OR (Sedentary Behaviors):ti,ab,kw OR (Sedentary Lifestyle):ti,ab,kw OR (Lifestyle, Sedentary):ti,ab,kw OR (Physical Inactivity):ti,ab,kw OR (Inactivity, Physical):ti,ab,kw OR (Lack of Physical Activity):ti,ab,kw OR (Sedentary Time):ti,ab,kw OR (Sedentary Times):ti,ab,kw OR (Time, Sedentary):ti,ab,kw

#13: #1 OR #2 OR #3 OR #4 OR #5 OR #6 OR #7 OR #8 OR #9 OR #10 OR #11 OR #12

#14: MeSH descriptor: [Telemedicine] explode all trees

#15: (smartphone application):ti,ab,kw OR (smartphone app):ti,ab,kw OR (mobile app):ti,ab,kw OR (mobile application):ti,ab,kw OR (app):ti,ab,kw OR (mobile phone):ti,ab,kw OR (smartphone*):ti,ab,kw OR (mobile device):ti,ab,kw OR (PDA):ti,ab,kw OR (tablet):ti,ab,kw OR (cell phone):ti,ab,kw OR (text message*):ti,ab,kw OR (sms):ti,ab,kw OR (short message service):ti,ab,kw OR (mobile health):ti,ab,kw OR (mHealth):ti,ab,kw OR (m-health):ti,ab,kw OR (internet):ti,ab,kw OR (telehealth):ti,ab,kw OR (telemedicine):ti,ab,kw OR (eHealth):ti,ab,kw OR (e-health):ti,ab,kw OR (iPod):ti,ab,kw OR (Fitbit):ti,ab,kw OR (Garmin):ti,ab,kw OR (Jawbone):ti,ab,kw OR (Nike):ti,ab,kw OR (ambulatory monitoring):ti,ab,kw OR (ambulatory assessment):ti,ab,kw OR (wireless technology):ti,ab,kw OR (accelerometer):ti,ab,kw OR (pedometer):ti,ab,kw OR (wearable*):ti,ab,kw OR (wearable activity tracker):ti,ab,kw

#16: (college student):ti,ab,kw OR (college students):ti,ab,kw OR (university student):ti,ab,kw OR (university students):ti,ab,kw OR (undergraduate):ti,ab,kw OR (undergraduates):ti,ab,kw OR (tertiary school student):ti,ab,kw OR (tertiary school students):ti,ab,kw OR (higher school student):ti,ab,kw OR (higher school students):ti,ab,kw

#17: #14 OR #15

#18: #13 AND #16 AND #17

Embase Searching:18

Included: 18; Search Name: e-health for pa and sb; Date Run:22/06/2022

No.	Query
#19	#13 AND #14 AND #17 AND #18
#18	#15 OR #16
#17	#1 OR #2 OR #3 OR #4 OR #5 OR #6 OR #7 OR #8 OR #9 OR #10 OR #11 OR #12

#16	'smartphone application':ab,ti OR 'smartphone app':ab,ti OR 'mobile app':ab,ti OR 'mobile application':ab,ti OR 'app':ab,ti OR 'mobile phone':ab,ti OR 'smartphone*':ab,ti OR 'mobile device':ab,ti OR 'pda':ab,ti OR 'tablet':ab,ti OR 'cell phone':ab,ti OR 'text message*':ab,ti OR 'sms':ab,ti OR 'short message service':ab,ti OR 'mobile health':ab,ti OR 'mhealth':ab,ti OR 'm-health':ab,ti OR 'internet':ab,ti OR 'telehealth':ab,ti OR 'telemedicine':ab,ti OR 'ehealth':ab,ti OR 'e-health':ab,ti OR 'ipod':ab,ti OR 'fitbit':ab,ti OR 'garmin':ab,ti OR 'jawbone':ab,ti OR 'nike':ab,ti OR 'ambulatory monitoring':ab,ti OR 'ambulatory assessment':ab,ti OR 'wireless technology':ab,ti OR 'accelerometer':ab,ti OR 'pedometer':ab,ti OR 'wearable*':ab,ti OR 'wearable activity tracker':ab,ti
#15	'telehealth'/exp
#14	'college student':ab,ti OR 'college students':ab,ti OR 'university student':ab,ti OR 'university students':ab,ti OR 'undergraduate':ab,ti OR 'undergraduates':ab,ti OR 'tertiary school student':ab,ti OR 'tertiary school students':ab,ti OR 'higher school student':ab,ti OR 'higher school students':ab,ti
#13	'randomized controlled trial':ab,ti OR 'randomized':ab,ti OR 'placebo':ab,ti
#12	'behavior, sedentary':ab,ti OR 'sedentary behaviors':ab,ti OR 'sedentary lifestyle':ab,ti OR 'lifestyle, sedentary':ab,ti OR 'physical inactivity':ab,ti OR 'inactivity, physical':ab,ti OR 'lack of physical activity':ab,ti OR 'sedentary time':ab,ti OR 'sedentary times':ab,ti OR 'time, sedentary':ab,ti
#11	'sedentary lifestyle'/exp
#10	'walk':ab,ti OR 'ambulation':ab,ti
#9	'walking'/exp
#8	'exercise, muscle stretching':ab,ti OR 'exercises, muscle stretching':ab,ti OR 'muscle stretching exercise':ab,ti OR 'static stretching':ab,ti OR 'stretching, passive':ab,ti OR 'passive stretching':ab,ti OR 'relaxed stretching':ab,ti OR 'stretching, relaxed':ab,ti OR 'static-passive stretching':ab,ti OR 'static passive stretching':ab,ti OR 'stretching, static-passive':ab,ti OR 'isometric stretching':ab,ti OR 'stretching, isometric':ab,ti OR 'active stretching':ab,ti OR 'stretching, active':ab,ti OR 'static-active stretching':ab,ti OR 'static active stretching':ab,ti OR 'stretching, static-active':ab,ti OR 'ballistic stretching':ab,ti OR 'stretching, ballistic':ab,ti OR 'dynamic stretching':ab,ti OR 'stretching, dynamic':ab,ti OR 'proprioceptive neuromuscular facilitation (pnf) stretching':ab,ti
#7	'stretching exercise'/exp
#6	'stamina, physical':ab,ti OR 'physical stamina':ab,ti OR 'endurance, physical':ab,ti
#5	'endurance'/exp

#4	'training, resistance':ab,ti OR 'strength training':ab,ti OR 'training, strength':ab,ti OR 'weight-lifting strengthening program':ab,ti OR 'strengthening program, weight-lifting':ab,ti OR 'strengthening programs, weight-lifting':ab,ti OR 'weight lifting strengthening program':ab,ti OR 'weight-lifting strengthening programs':ab,ti OR 'weight-lifting exercise program':ab,ti OR 'exercise program, weight-lifting':ab,ti OR 'exercise programs, weight-lifting':ab,ti OR 'weight lifting exercise program':ab,ti OR 'weight-lifting exercise programs':ab,ti OR 'weight-bearing strengthening program':ab,ti OR 'strengthening program, weight-bearing':ab,ti OR 'strengthening programs, weight-bearing':ab,ti OR 'weight bearing strengthening program':ab,ti OR 'weight-bearing strengthening programs':ab,ti OR 'weight-bearing exercise program':ab,ti OR 'exercise program, weight-bearing':ab,ti OR 'exercise programs, weight-bearing':ab,ti OR 'weight bearing exercise program':ab,ti OR 'weight-bearing exercise programs':ab,ti
#3	'resistance training'/exp
#2	'exercise':ab,ti OR 'exercises':ab,ti OR 'physical activity':ab,ti OR 'activities, physical':ab,ti OR 'activity, physical':ab,ti OR 'physical activities':ab,ti OR 'exercise, physical':ab,ti OR 'exercises, physical':ab,ti OR 'physical exercise':ab,ti OR 'physical exercises':ab,ti OR 'acute exercise':ab,ti OR 'acute exercises':ab,ti OR 'exercise, acute':ab,ti OR 'exercises, acute':ab,ti OR 'exercise, isometric':ab,ti OR 'exercises, isometric':ab,ti OR 'isometric exercises':ab,ti OR 'isometric exercise':ab,ti OR 'exercise, aerobic':ab,ti OR 'aerobic exercise':ab,ti OR 'aerobic exercises':ab,ti OR 'exercises, aerobic':ab,ti OR 'exercise training':ab,ti OR 'exercise trainings':ab,ti OR 'training, exercise':ab,ti OR 'trainings, exercise':ab,ti
#1	'physical activity'/exp

Web of Science Searching:

Included: 417; Search Name: e-health for pa and sb; Date Run: 22/06/2022

TS=(smartphone application* OR smartphone app* OR mobile app* OR mobile application* OR app* OR mobile phone* OR smartphone* OR mobile device* OR PDA* OR tablet* OR cell phone* OR text message* OR sms OR short message service* OR mobile health* OR mHealth* OR m-health* OR internet* OR telehealth* OR telemedicine* OR eHealth* OR e-health* OR iPod* OR Fitbit* OR Garmin* OR Jawbone* OR Nike* OR ambulatory monitor* OR ambulatory assessment* OR wireless technolog* OR accelerometer* OR pedometer* OR wearable* OR wearable activity tracker*)

TS=(Exercise* OR Physical Activit* OR Activit*, Physical OR Activit*, Physical OR Physical Activit* OR Exercise*, Physical OR Exercise*, Physical OR Physical Exercise* OR Physical Exercise* OR Acute Exercise* OR Exercise*, Acute OR Exercise*, Isometric OR Isometric Exercise* OR Exercise*, Aerobic OR Aerobic Exercise* OR Exercise*, Aerobic OR Exercise Training* OR Training*, Exercise OR Resistance Training OR Training, Resistance OR Strength Training OR Training, Strength OR Weight-Lifting Strengthening Program* OR Strengthening Program*, Weight-Lifting OR Weight-Lifting Strengthening Program* OR Weight-Lifting Exercise Program* OR Exercise Program*, Weight-Lifting OR Weight-Lifting Exercise Program* OR Weight-Bearing Strengthening Program* OR Strengthening Program*, Weight-Bearing OR Weight-Bearing Strengthening Program* OR Weight-Bearing Exercise Program* OR Exercise Program*, Weight-Bearing OR Weight-Bearing Exercise Program* OR
Physical Endurance OR Stamina, Physical OR Physical Stamina OR Endurance, Physical OR
Muscle Stretching Exercises OR Exercise, Muscle Stretching OR Exercises, Muscle Stretching OR Muscle Stretching Exercise OR Static Stretching OR Stretching, Passive OR Passive Stretching OR Relaxed

Stretching OR Stretching, Relaxed OR Static-Passive Stretching OR Static Passive Stretching OR Stretching, Static-Passive OR Isometric Stretching OR Stretching, Isometric OR Active Stretching OR Stretching, Active OR Static-Active Stretching OR Static Active Stretching OR Stretching, Static-Active OR Ballistic Stretching OR Stretching, Ballistic OR Dynamic Stretching OR Stretching, Dynamic OR Proprioceptive Neuromuscular Facilitation (PNF) Stretching OR walk* OR ambulation* OR Sedentary Behavior OR Behavior, Sedentary OR Sedentary Behaviors OR Sedentary Lifestyle OR Lifestyle, Sedentary OR Physical Inactivity OR Inactivity, Physical OR Lack of Physical Activity OR Sedentary Time OR Sedentary Times OR Time, Sedentary)

TS= (college student* OR university student* OR undergraduate* OR tertiary school student* OR higher school student*)

TS= (random* controlled trial OR random* OR placebo)

ProQuest Searching:

Included: 94; Search Name: e-health for pa and sb; Date Run: 22/06/2022

ab(("smartphone application*" OR "smartphone app*" OR "mobile app*" OR "mobile application*" OR app* OR "mobile phone*" OR smartphone* OR "mobile device*" OR PDA* OR tablet* OR "cell phone*" OR "text message*" OR sms OR "short message service*" OR "mobile health*" OR mHealth* OR m-health* OR internet* OR telehealth* OR telemedicine* OR eHealth* OR e-health* OR iPod* OR Fitbit* OR Garmin* OR Jawbone* OR Nike* OR "ambulatory monitor*" OR "ambulatory assessment*" OR "wireless technolog*" OR accelerometer* OR pedometer* OR wearable* OR "wearable activity tracker*")) AND ab((Exercise* OR "Physical Activit*" OR "Activit*, Physical" OR "Activit*, Physical" OR "Physical Activit*" OR "Exercise*, Physical" OR "Exercise*, Physical" OR "Physical Exercise*" OR "Physical Exercise*" OR "Acute Exercise*" OR "Exercise*, Acute" OR "Exercise*, Isometric" OR "Isometric Exercise*" OR "Exercise*, Aerobic" OR "Aerobic Exercise*" OR "Exercise*, Aerobic" OR "Exercise Training*" OR "Training*, Exercise" OR " Resistance Training" OR "Training, Resistance" OR "Strength Trainin" g OR "Training, Strength" OR "Weight-Lifting Strengthening Program*" OR "Strengthening Program*, Weight-Lifting" OR "Weight-Lifting Strengthening Program*" OR "Weight-Lifting Exercise Program*" OR "Exercise Program*, Weight-Lifting" OR "Weight-Lifting Exercise Program*" OR "Weight-Bearing Strengthening Program*" OR "Strengthening Program*, Weight-Bearing" OR "Weight-Bearing Strengthening Program*" OR "Weight-Bearing Exercise Program*" OR "Exercise Program*, Weight-Bearing" OR "Weight-Bearing Exercise Program*" OR "Physical Endurance OR Stamina, Physical" OR "Physical Stamina OR Endurance, Physical" OR "Muscle Stretching Exercises" OR "Exercise, Muscle Stretching" OR "Exercises, Muscle Stretching" OR "Muscle Stretching Exercise" OR "Static Stretching" OR "Stretching, Passive" OR "Passive Stretching" OR "Relaxed Stretching" OR "Stretching, Relaxed" OR "Static-Passive Stretching" OR "Static Passive Stretching" OR "Stretching, Static-Passive" OR "Isometric Stretching" OR "Stretching, Isometric OR Active Stretching" OR "Stretching, Active" OR "Static-Active Stretching" OR "Static Active Stretching" OR "Stretching, Static-Active" OR "Ballistic Stretching" OR "Stretching, Ballistic" OR "Dynamic Stretching" OR "Stretching, Dynamic" OR "Proprioceptive Neuromuscular Facilitation (PNF) Stretching" OR walk* OR ambulation* OR "Sedentary Behavior" OR "Behavior, Sedentary" OR "Sedentary Behaviors" OR "Sedentary Lifestyle" OR "Lifestyle, Sedentary" OR "Physical Inactivity" OR "Inactivity, Physical" OR "Lack of Physical Activity" OR "Sedentary Time" OR "Sedentary Times" OR "Time, Sedentary")) AND ab(("college student*" OR "university student*" OR "undergraduate*" OR "tertiary school student*" OR "higher school student*")) AND ab(("random* controlled trial" OR random* OR placebo))
