# Supplementary figures and images for: The Effectiveness of E-Health Interventions Promoting Physical Activity and Reducing Sedentary Behavior in College Students: A Systematic Review and Meta-Analysis of Randomized Controlled Trials
Source: Int J Environ Res Public Health. 2022 Dec 25;20(1):318. doi: 10.3390/ijerph20010318 (PMC9819541; doi:10.3390/ijerph20010318)

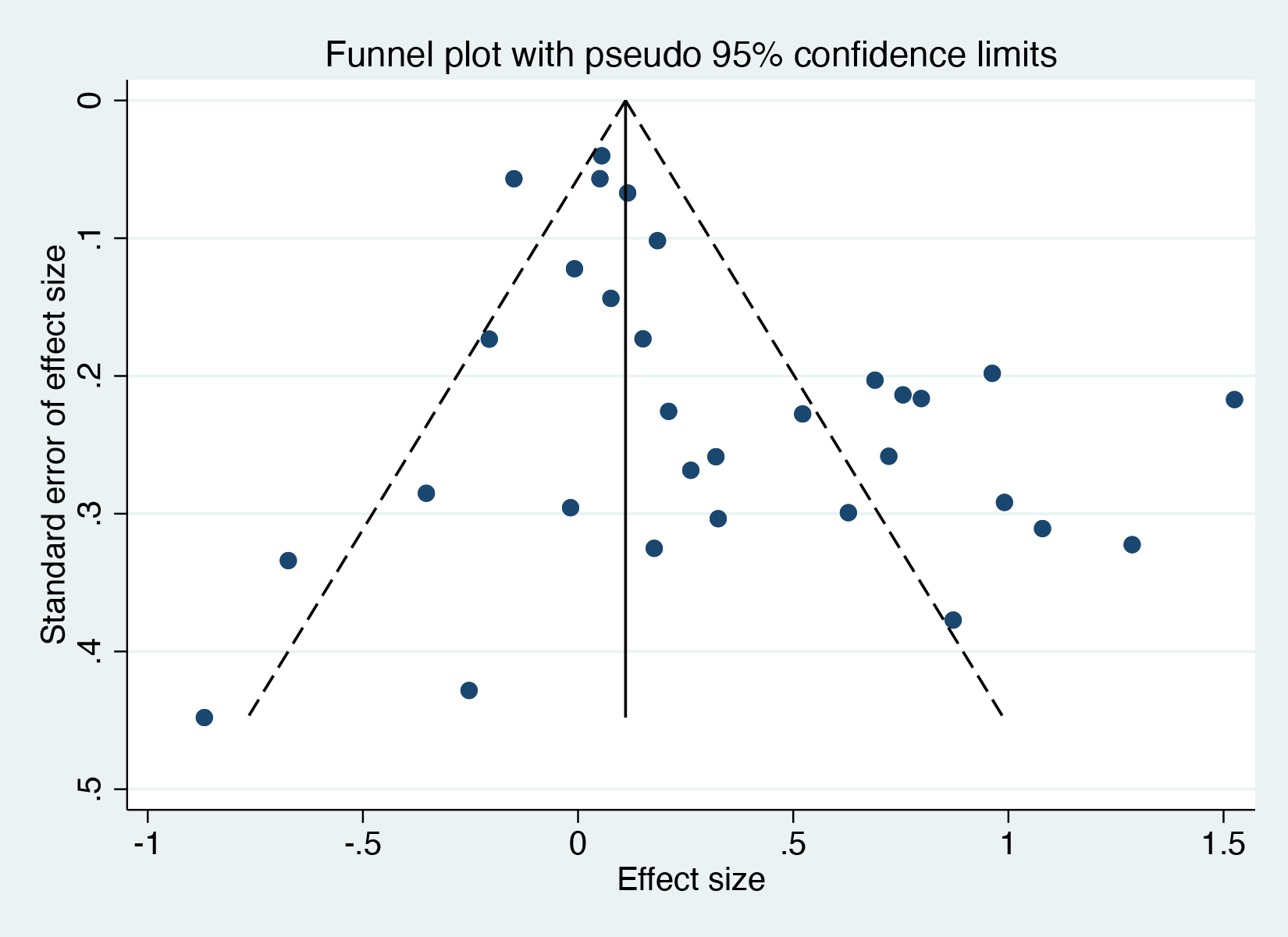

Supplement: Supplementary file 1 [file ijerph-20-00318-s001.zip › Figure S1 Funnel plot for PA (post-intervention).tif]

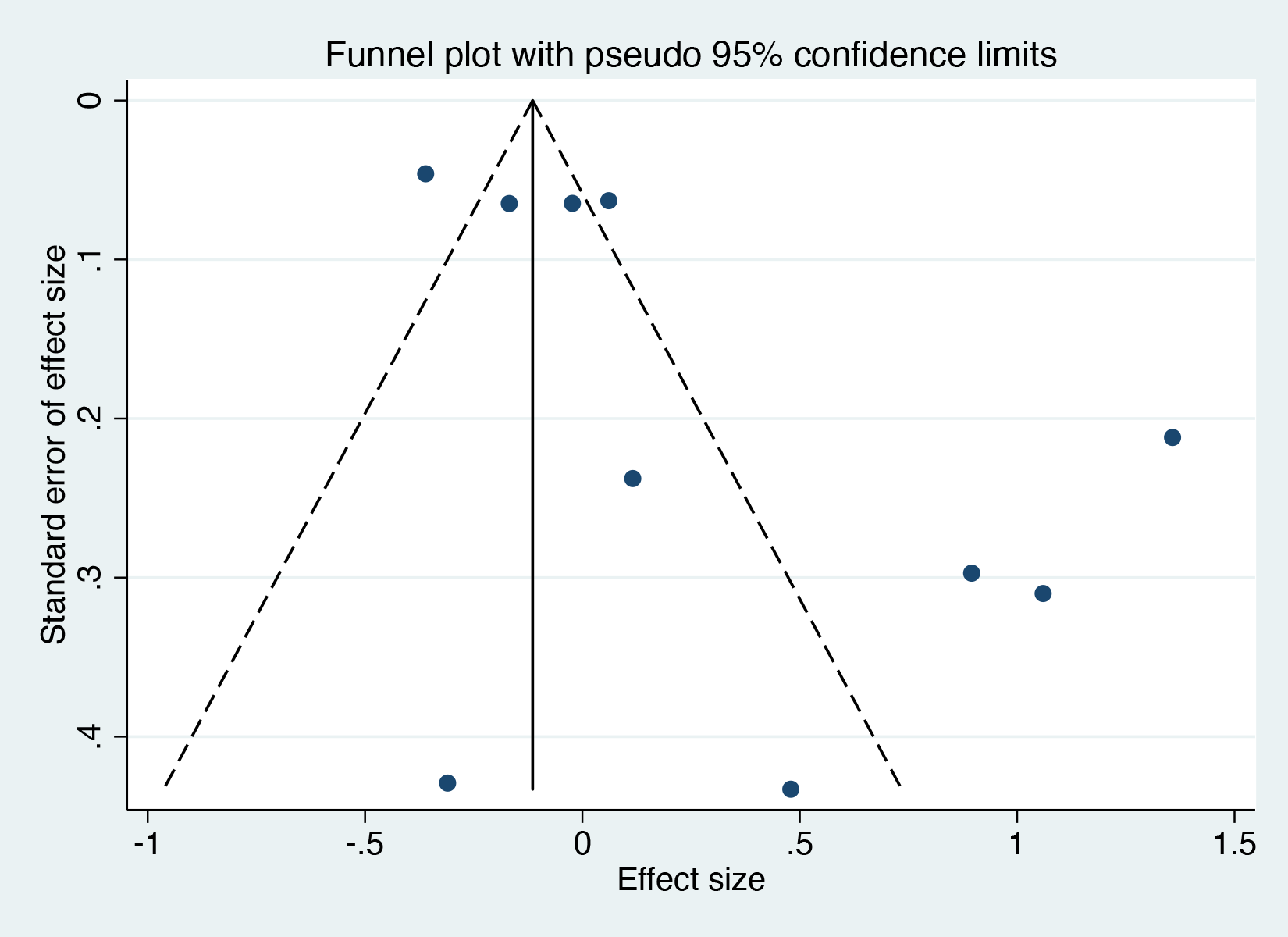

Supplement: Supplementary file 1 [file ijerph-20-00318-s001.zip › Figure S2 Funnel plot for follow-up.tif]

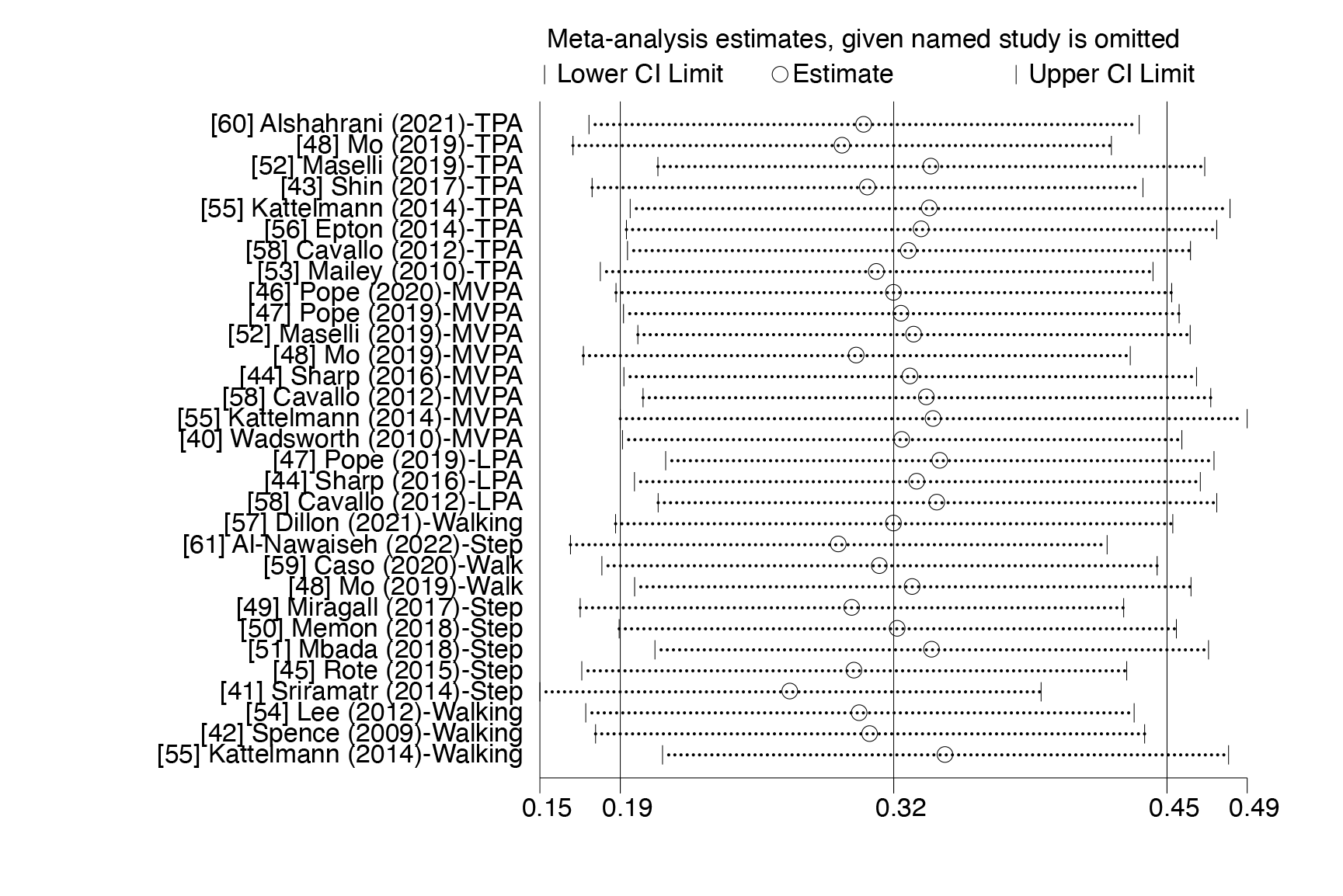

Supplement: Supplementary file 1 [file ijerph-20-00318-s001.zip › Figure S3 Sensitivity analysis (post-intervention).tif]

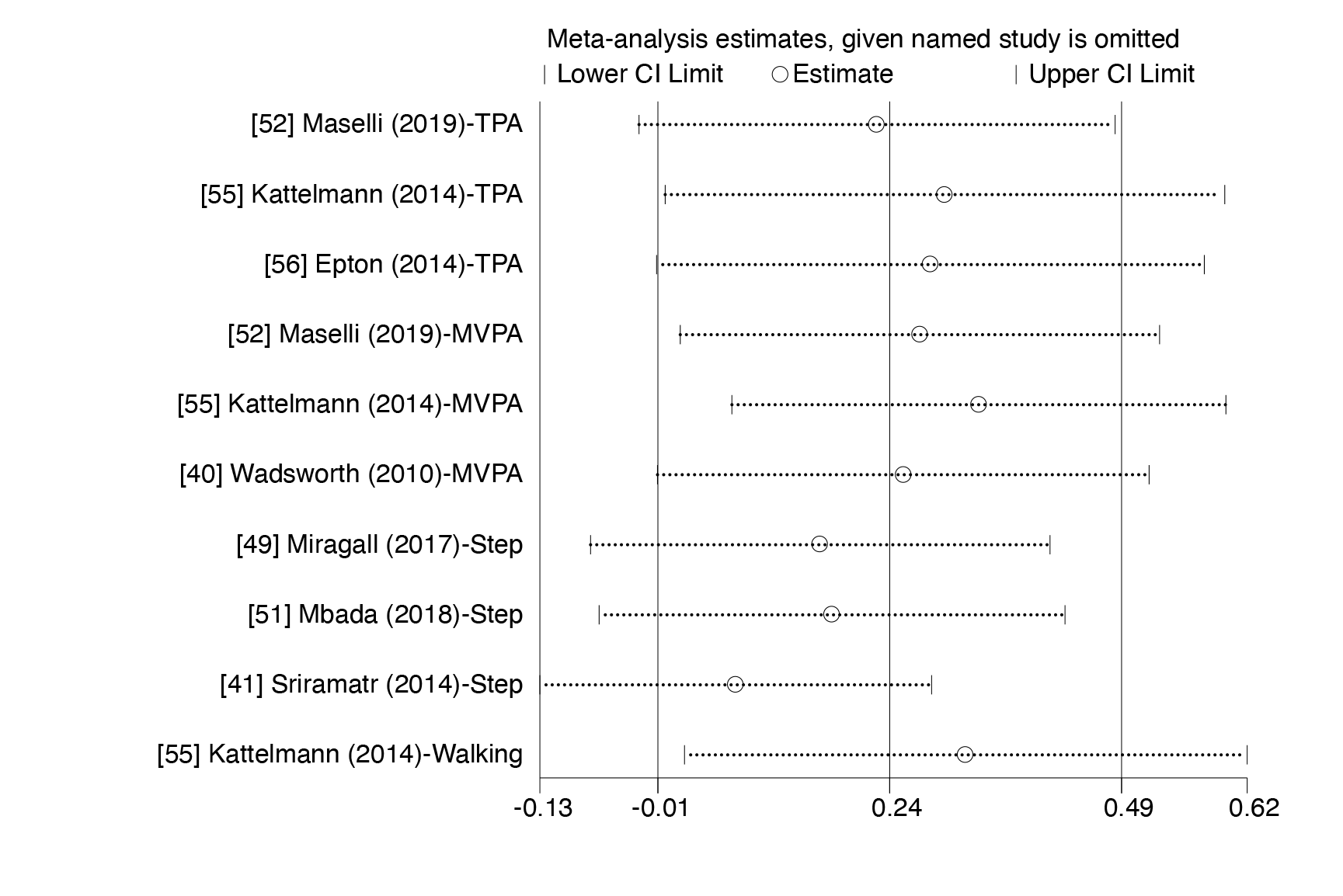

Supplement: Supplementary file 1 [file ijerph-20-00318-s001.zip › Figure S4 Sensitivity analysis of PA(follow-up).tif]

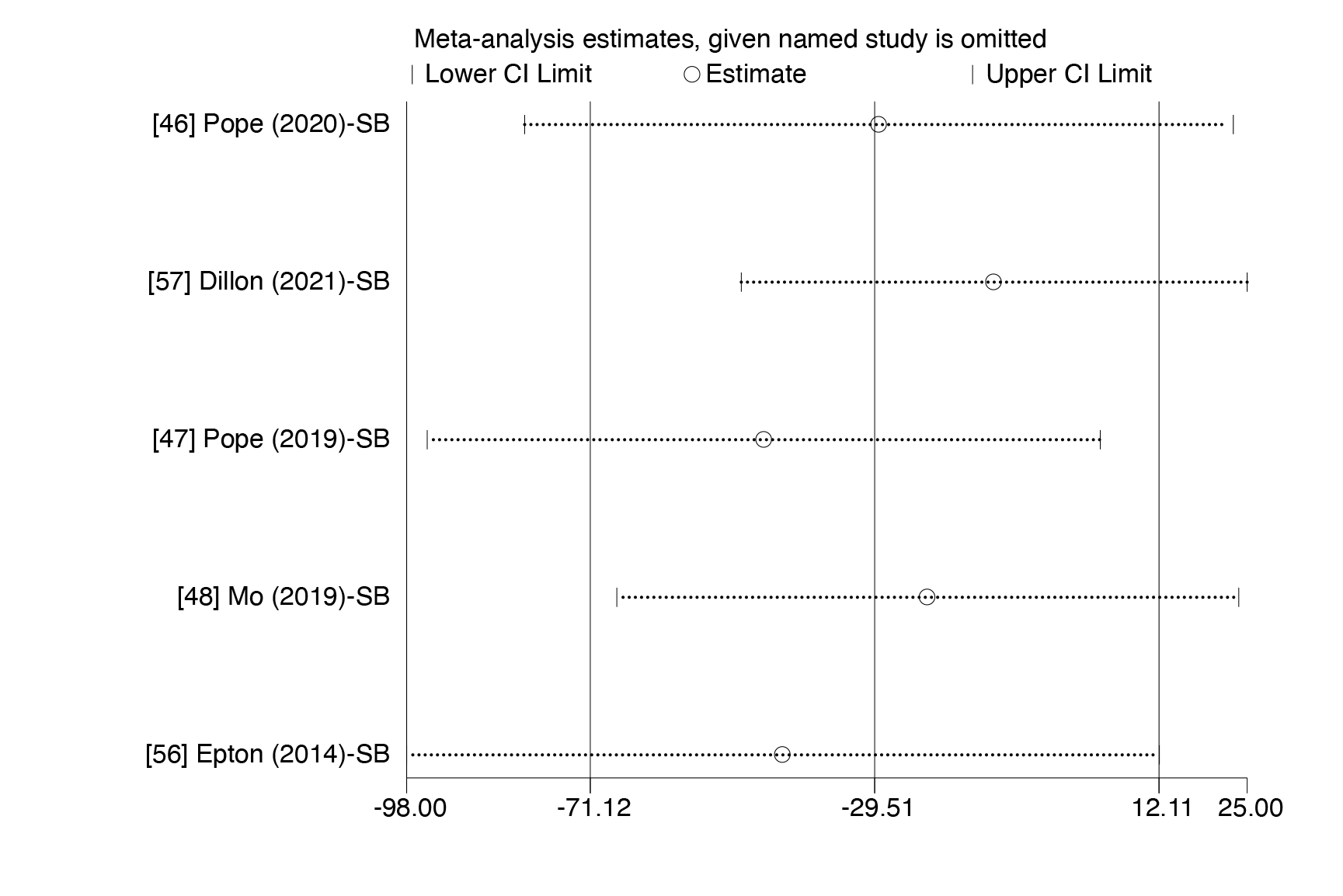

Supplement: Supplementary file 1 [file ijerph-20-00318-s001.zip › Figure S5 Sensitivity analysisú¿SBú⌐.tif]
